# A novel method for utilizing dichoptic attention tasks in amblyopic training

**DOI:** 10.1016/j.mex.2022.101829

**Published:** 2022-08-22

**Authors:** Chuan Hou

**Affiliations:** The Smith-Kettlewell Eye Research Institute, United States

**Keywords:** Amblyopia, Strabismus, Visual search, Feature counting, Selective visual attention

## Abstract

There is converging evidence of attention deficits in individuals with amblyopia. It has been reported that selective visual attention is allocated preferentially toward the non-amblyopic fellow eye in strabismic ambloypes. This attention allocation bias between the eyes is found related to visual suppression in amblyopia. In this study, I introduced a novel method, which uses dichoptic attention tasks to train individuals with amblyopia and has been demonstrated alleviating visual suppression and improving visual functions while reducing interocular attention bias in adults with amblyopia. The method consists of the following components:

• The training tasks include three attention factors (searching, counting and cueing) to implement selective visual attention to the amblyopic eye dichoptically.
• With a dichoptic approach, the targets are presented to the amblyopic eye while the distractors are simultaneously presented to the fellow eye. The tasks are to search and count the targets that are presented to the cued eye among the distractors.
• The training stimuli avoid typical contrast sensitivity-based tasks, and the targets are highly visible allowing them to be seen by the amblyopic eye with poor visual acuity.

## Graphical abstract

**Figure:**
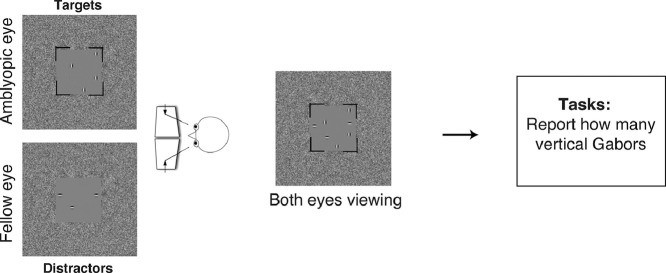

**Specifications table**

**Table:** 

Subject Area:	Neuroscience
More specific subject area:	Behavioral training in amblyopia
Method name:	Dichoptic attention task as a method for behavioral training in amblyopia
Name and reference of original method:	None
Resource availability:	The script in Matlab (free downloadable software) is available on GitHub

## Background

Amblyopia (‘lazy eye’) is the leading cause of monocular vision loss in children and affects about 3-5% of the population [1]. The standard clinical treatment is to patch the non-amblyopic fellow eye to promote the use of the amblyopic eye. This treatment benefits most amblyopic children with 20-25% recurrence following the treatment [2], [3], leaving about 1/3 of affected children that persists into adulthood [4]. There is no available clinical treatment for adults with amblyopia.

Long-term and chronic visual suppression to the visual input from the non-preferred eye is a key factor in developing amblyopia [5], as well as a critical barrier to treat amblyopia [6]. In addition to visual acuity loss and binocular function interruption, recent studies reveal that individuals with amblyopia also exhibit attention deficits [7], [8], [9], [10]. It has been reported that selective visual attention is preferentially allocated toward the non-amblyopic fellow eye in strabismic ambloypes [10], [11], and that a degraded attentional modulation in V1 neurons to the visual input from the amblyopic eye of strabismic amblyopes correlates to the degree of interocular suppression and the depth of amblyopia measured behaviorally [10]. In this study, I introduced a novel method that uses dichoptic attention tasks to train individuals with amblyopia, which has been demonstrated reducing attention allocation bias between the eyes (referred to as “interocular attention bias”) and alleviating interocular suppression accompanied with improvements of visual functions in adults with amblyopia [12].

## Method design

**Fig. 1:**
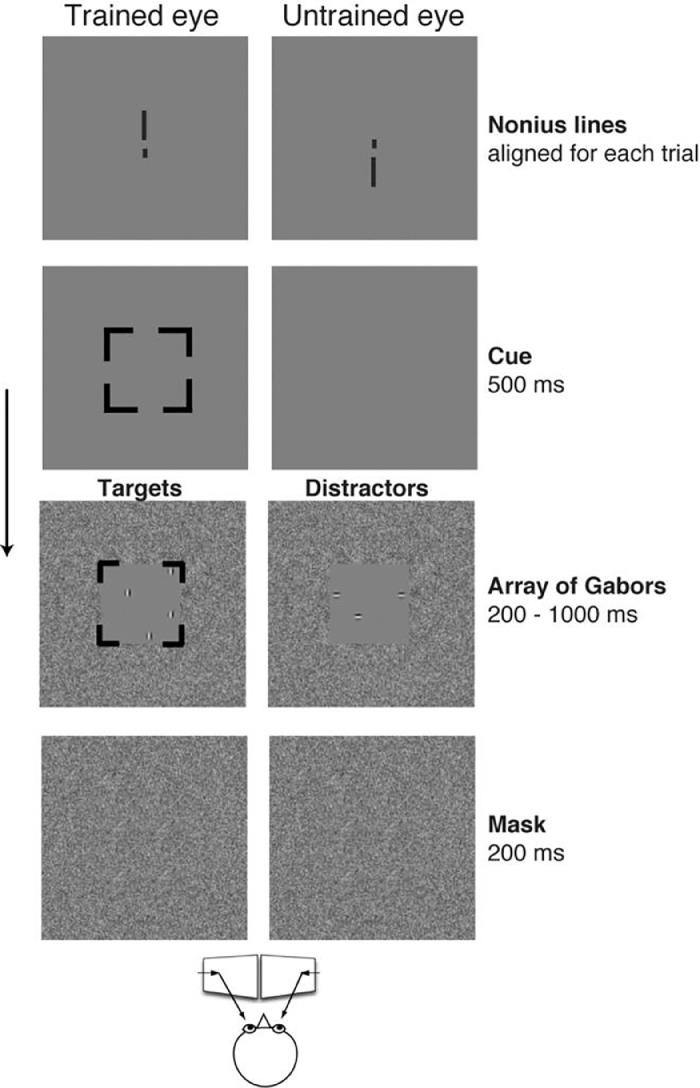
Illustration of method using dichoptic attention tasks in amblyopic training and the temporal sequence of a given trial in training sessions. Adapted from the related validation article [1] that used this method in amblyopic training in adults.

As seen in Fig. 1, the dichoptic attention-training stimuli include three key factors (searching, counting and cueing) to implement selective visual attention to the amblyopic eye (i.e., trained eye) under dichoptic viewing through a mirror stereoscope, in which both the horizontal and vertical deviations in strabismic amblyopes are adjusted by mirrors to align the nonius lines under the best optical correction. The tasks are to search and count the vertical Gabors (targets) that are presented in the cued eye (i.e., trained eye) among the horizontal Gabors (distractors) that are simultaneously presented in the uncued eye (i.e., untrained eye). The details of the method design are explained below. Firstly, *searching* for targets among distractors requires significant attentional focus [13], [14], [15]. Simultaneously presenting distractors in the fellow eye may induce suppression process to the visual input from the fellow eye by top-down control, according to the framework of attention and distractor suppression [15], [16]. A recent fMRI study [17] reveals that distractors in visual search stimuli are suppressed starting as early as the primary visual cortex (V1). Therefore, the arrangement of the targets in the amblyopic eye and the distractors in the fellow eye in the training stimuli implements selective visual attention to the amblyopic eye *dichoptically* and reverses the habitual condition in amblyopia that commonly dominates attention by the fellow eye. This design is expected to induce distractor suppression process to visual input from the fellow eye while allocating attention to visual input from the trained amblyopic eye for a target search, which is hoped to reduce interocular attention bias that is typically found in strabismuc amblyopes [10], [11].

Secondly, *feature counting*, particularly when counting large set-sizes of Gabors (e.g., Gabor set-sizes > 5) [18], requires rapid shifts in attention and involves visual attention processes [19], [20] and activates neural activity in intraparietal sulcus (IPS) [21], [22], a region known to be involved in visual attention [23]. Our previous studies [24], [25] and others [7] have showed that feature counting with large set-sizes of Gabors (n> 5) is impaired in the amblyopic eye of adults with amblyopes, and that it is further impaired under conditions of interocular suppression [24]. Therefore, feature counting is used in the training stimuli, in which a random array of Gabor patches with different orientations presents to each eye and the tasks are only to count the number of vertical Gabors (targets) that are presented to the amblyopic eye among distractors (horizontal Gabors) that are presented to the fellow eye. Selective visual attention with trained task (i.e., searching/counting performance) and with untrained task (attentional modulation in population neurons in V1 and IPS) have been measured to confirm whether training improves selective attention to the visual input from the amblyopic eye [12].

Thirdly, a *perceptual cue* guides attention to the trained eye, which helps observers to capture the new target in advance before the stimuli [26], [27]. Our own pilot study [28] has examined the effect of cueing the amblyopic eye in humans with a valid and neutral cue, similar to other studies in human [29] and monkey [30] amblyopes, and has shown that cueing the amblyopic eye improves task performance. In the training protocol of validation study [12], we used 100% valid cue to the trained eye and arranged 90% of the trials cueing the amblyopic eye and with only 10% of the trials cueing the fellow eye in a random order within a block. It is worth noting that each factor (searching, counting, or cueing) described above could independently implement selective visual attention [13], [14], [15], [27]. It is expected that combining these three attention factors into training tasks requires significant attentional efforts from the amblyopic eye and expects to improve attention deployment considerably in the amblyopic eye, and thus to reduce interocular attention bias. The reduction of interocular attention bias by training has been confirmed in the validation study [12]. Lastly, the stimuli avoid low-level visual features (e.g., low contrast and high spatial frequency) in the tasks and make the counting elements (Gabors) highly visible at high contrast (≥ 25%) and low spatial frequency (2 cycle /deg) allowing them to be seen by the amblyopic eye with poor visual acuity. The illustration and procedure of the method for utilizing dichoptic attention tasks in amblyopic training are shown in Fig. 1.

In Fig. 1, for a given trial with targets (4 vertical Gabors) in the trained eye and distractors (3 horizontal Gabors) in the untrained eye under dichoptic viewing through a mirror stereoscope, a total of 7 patches including both vertical and horizontal Gabors are seen during dichoptic perception. The Gabors are highly visible (above 25% contrast at 2 cpd of spatial frequency) to ensure that the amblyopic eye can see the Gabor patches. The tasks are to search and count the vertical Gabors. The temporal sequence of a given trial in training sessions as the following: A central fixation point and nonius lines appear prior to the initiation of all trials, to ensure that the mirror stereoscope remains properly aligned. A 500 ms-valid attentive cue (square) precedes the stimuli to indicate which eye would see the targets. Then, a random array of Gabor patches with different orientations will be simultaneously presented to the two eyes. The stimuli display for 200-1000 ms (depending on the stages of training sessions) followed by a 200 ms noise mask. The trials are self-initiated and the participants are requested to respond as accurately as possible with no time limit and no feedback is given.

## Training protocols

The contrasts of Gabor patches in the two eyes are equal (35% contrast in each eye) across training sessions, in which the level of attention required is incremented by progressively decreasing stimulus display duration from the initial session at 1000 ms to the end session at 200 ms. In the training of validation study [12], the stimuli were presented at a viewing distance of 85 cm on a pair of Sony Trinitron Multiscan G400 CRT monitors with a frame rate of 85 Hz. An adjustable mirror stereoscope was used to combine the left-eye and right-eye views into a single view. The participants were required to come in to the lab for training for about 2 visits per week, 2 hours per visit, for 2 months (in total of 16 visits, ∼7000 trials of repetitive practice). This amount of training was chosen because observers have shown no further improvement after 7000 trials [31].

## Method validation

Using dichoptic attention tasks described in this study, 13 adults with amblyopia (6 anisometropic and 7 strabismic or mixed anisometropic/strabismic), including 4 participants who did not complete 16 sessions, showed improvement of attentional deployment in the amblyopic eye, reduction of interocular attention bias, alleviation of interocular suppression and the visual function improvement. All data that validate the method have been provided in the related research article, titled “Perceptual learning with dichoptic attention tasks improves attentional modulation in V1 and IPS neurons and reduces interocular suppression in human adults with amblyopia” [12].

## Conclusions

In summary, a novel method that uses dichoptic attention tasks to train individuals with amblyopia is introduced in this study. This method has been demonstrated alleviating interocular suppression and improving visual functions when improving selective visual attention to the amblyopic eye by training. This method provides new insights that training with a dichoptic approach that incorporates attention demand tasks in the amblyopic eye might be an effective way of treating amblyopia.

## CRediT author statement

**Chuan Hou:** Conceptualization, Design of Method, Validity tests, Writing, Reviewing and Editing.

## Declaration of Competing Interest

The author declares no competing financial interests or personal relationships that could have appeared to influence the work reported in this paper.

## Data Availability

Data will be made available on request.
